# In Vivo and Cadaver Studies of the Canalicular/Lacrimal Sac Mucosal Folds

**DOI:** 10.1155/2016/3453908

**Published:** 2016-05-08

**Authors:** Yongsheng You, Jing Cao, Xiaogang Zhang, Wencan Wu, Tianlin Xiao, Yunhai Tu

**Affiliations:** ^1^Eye Center of The Second Affiliated Hospital, School of Medicine, Zhejiang University, 88 Jiefang Road, Hangzhou, Zhejiang 310009, China; ^2^Eye Hospital of Wenzhou Medical University, 270 West Xueyuan Road, Wenzhou, Zhejiang 325027, China

## Abstract

*Purpose.* The study aimed to investigate canalicular/lacrimal sac mucosal folds (CLS-MFs) in vivo and in cadavers in order to explore their functional roles in the lacrimal drainage system.* Method.* The observations of CLS-MFs in vivo were performed on 16 patients with chronic dacryocystitis after undergoing an endonasal endoscopic dacryocystorhinostomy (EE-DCR). The lacrimal sacs and common canaliculi of 19 adult cadavers were dissected. The opening/closing of an orifice and mucosal fold was recorded. All of the specimens were subjected to a histological examination.* Results.* The upper and lower lacrimal canaliculi in all of the samples united to form a common canaliculus that opened to the lacrimal sac. CLS-MFs were observed in 10 of the 16 patients (62.5%) and 9 of the 19 cadavers (47.4%). The orifices or mucosal folds could be opened or closed when related muscles contracted or relaxed. Histological sections showed a mucosal fold at one side of an orifice.* Conclusion.* Common canaliculus is the most common type that the canaliculus opens to lacrimal sac. CLS-MFs exist in a certain ratio that can be opened/closed with the movement of the orifices. They may be involved in the drainage of tears or the pathogenesis of acute dacryocystitis or lacrimal sac mucocele.

## 1. Introduction

Since 1797 when the Rosenmuller valve was first described [1], there were only a few publications describing the CLS-MFs and their functional roles [2–4]. Zoumalan et al. reported that 74 (59.7%) in 124 lacrimal systems had some variation of mucosal folds, and the remaining 50 (40.3%) had no visible mucosal fold [4]. Kakizaki et al. showed that an orifice open/close could be watched at the joint of the lacrimal canaliculus and the lacrimal sac following a blink under the observation of a nasal endoscope after a dacryocystorhinostomy (DCR) [5]. In clinic, one puzzling phenomenon is that it is impossible to press the mucous or purulent discharge from a lacrimal sac over to the puncta in the patients with acute dacryocystitis or a lacrimal sac mucocele. Those clinical features suggested that a valve or a one-way valve may exist at the canalicular/lacrimal sac junction [6–8]. However, currently, the existence of a valve or a mucosal fold at the canaliculus entrance into the lacrimal sac remains a controversial issue; moreover, the reports have no histological evidence of a real valve at the canaliculus entrance into the lacrimal sac [2]. Recently, we observed CLS-MFs in 16 patients with chronic dacryocystitis after endonasal endoscopic-DCRs (EE-DCRs). Meanwhile, we dissected a group of lacrimal drainage systems from 19 adult cadavers to investigate the mucosal folds in vivo and in cadavers.

## 2. Materials and Methods

Video clips and photographs of CLS-MFs in vivo were obtained from 16 patients with chronic dacryocystitis after EE-DCRs. This study complied with the tenets of the Declaration of Helsinki. It was approved by our hospital ethics committee, and consent forms were obtained from all of the patients. The patients were selected as the study subjects due to their wide open ostia and visible mucosal folds. The open/close of the orifices or mucosal folds was recorded with the aid of a nasal endoscope connected to a digital camera (Storz, Germany) during a follow-up period of 1 to 8 months after operation.

The 19 cadavers used for the study were provided by the Department of Anatomy of Wenzhou Medical University with the permission of the institutional review board. Cadavers were excluded from the study if there were prior dissections or anatomical damage to the structures of the eyelid. An ophthalmic operating microscope was used for the dissection via a standard external DCR incision, followed by a blunt dissection to expose the sac in the lacrimal fossa. After incising the medial canthal tendon to allow for better exposure, the lacrimal sac (including a portion of the lacrimal canaliculus) was dissected. Scissors were used to open the lacrimal sac along the longitudinal axis of the medial wall to expose the mucosal fold and orifice. The orifice open/close was recorded when the muscles around the sac were relaxed or stretched using forceps.

The histological examination consisted of a conventional pathological biopsy and 5 *μ*m sequential sections along the longitudinal axis of the lacrimal sac. One out of every five pieces was examined using hematoxylin and eosin (H&E) staining.

## 3. Results and Discussion

### 3.1. Results

**Figure 1:**
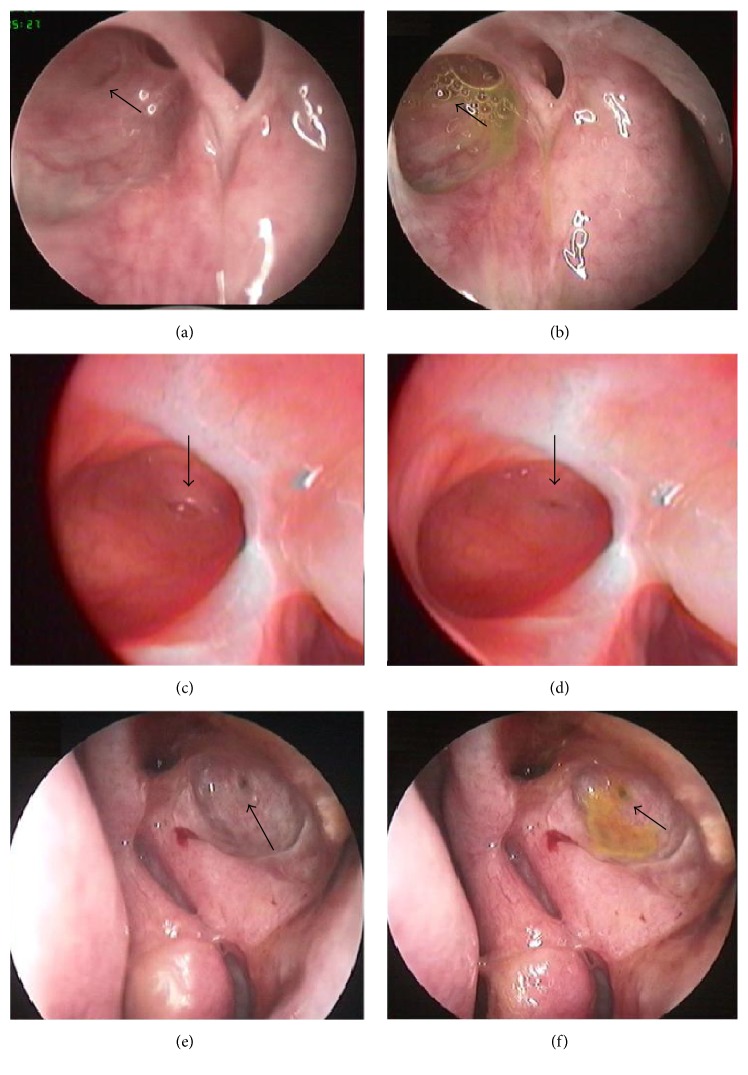
Observation of the mucosal folds in the EE-DCR patients. (a) and (b) show anterior mucosal folds (right nose and black arrow) with and without a fluorescein appearance test (the latter also with a few air bubbles). (c) displays an upper mucosal fold (right nose) with an open orifice in a circle area (black arrow) during the eyelid closing, and (d) displays an incompletely closed orifice (right nose and black arrow) during the eyelid opening. (e) and (f) display the orifices (left nose) without remarkable mucosal folds (the latter with a positive fluorescein appearance test).

Sixteen patients following a successful EE-DCR showed a wide open ostium. Mucosal folds were found in 10 out of 16 cases (62.5%) (Figures 1(a)–1(d)), and the remaining six cases (37.5%) (Figures 1(e) and 1(f)) had no remarkable mucosal fold. Mucosal folds were located anteriorly (Figures 1(a) and 1(b)) in four cases and posteriorly, inferiorly, and superiorly (Figures 1(c) and 1(d)) in two cases, respectively.  Figure 1(c) shows an opened orifice and mucosal fold during the eyelid closing, and Figure 1(d) presents an incompletely closed orifice during the eyelid opening.

**Figure 2:**
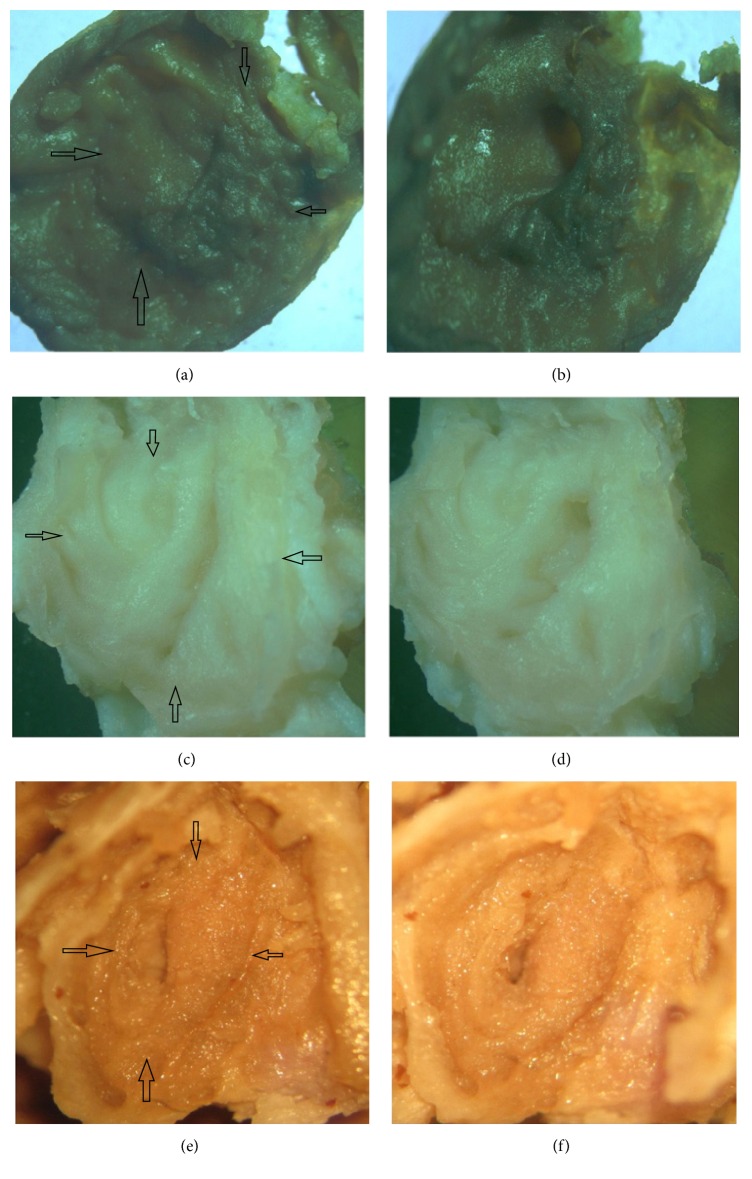
Orifice open/close under the relaxation/stretch of the muscles around the lacrimal sac. (a), (c), and (e) show that the orifices are closed when the related muscles are stretched. (b), (d), and (f) show that the orifices are opened when the muscles are relaxed. It should be noted that there is always a circle area (arrows) around an orifice.

Nineteen lacrimal sac specimens were successfully dissected. The general anatomical study showed all specimens with a common canaliculus entrance into the lacrimal sac (19/19, 100%), and 9 of the 19 samples (47.4%) had mucosal folds around the canaliculus entrance into the lacrimal sac. The orifices were closed (Figures 2(a), 2(c), and 2(e)) when the muscles around the lacrimal sac were stretched and were opened when the muscles were relaxed (Figures 2(b), 2(d), and 2(f)). It was noted that when the muscles were relaxed or stretched, a circle area always existed around the mucosal fold or orifice (black arrows, Figures 2(a), 2(c), and 2(e)).

**Figure 3:**
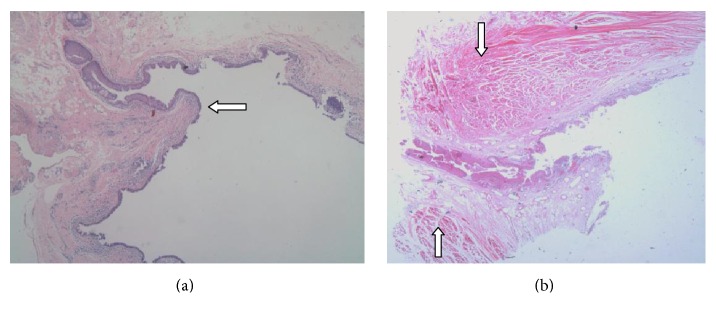
H&E staining of canalicular entrance into the lacrimal sac. (a) shows a protruding mucosal fold at the entrance of a common canaliculus to the lacrimal sac, which is covered with epithelial cells of saccus lacrimalis (arrow). The arrows in (b) show many bundles of circular and longitudinal muscles around this region.

Histological sections confirmed the existence of mucosal folds in cadavers, which presented with a protrusion at one side of the orifice (Figure 3(a), arrow). Many bundles of circular and longitudinal muscles were around the area (Figure 3(b), arrows). Figure 3(a) also shows the common canaliculus lined with stratified squamous epithelial cells and the lacrimal sac lined with double-layered columnar epithelial cells.

### 3.2. Discussion

Our study showed all specimens had a common canaliculus that opened to the lacrimal sac (100% of prevalence), which was higher than the 90% of the average prevalence reported in the literature [4, 9]. Studies by Orhan et al. showed that the upper lacrimal canaliculus and lower lacrimal canaliculus were opened to the lacrimal sac in the following three types: Type A, Type B, and Type C [10–12]. In Type A, the upper and lower canaliculi unite before opening to the lacrimal sac and form a common canaliculus. In Type B, the upper and lower canaliculi unite at the wall of the lacrimal sac and open to the lacrimal sac via a common hole. In Type C, the upper and lower canaliculi open to the lacrimal sac separately. Orhan et al. reported that Type A, Type B, and Type C were observed in 85%, 5%, and 10% cases, respectively [10]. B. Yazici and Z. Yazici performed dacryocystographies in 2000 that showed common canaliculi in 321 (94.1%) out of 341 lacrimal drainage systems, 3.8% with upper and lower canaliculi joined at the wall of the lacrimal sac, and only 7 (2.0%) with the upper and lower canaliculi entering the sac separately [9]. In 2011, Zoumalan et al. reported that 123 (99.2%) out of 124 lacrimal systems demonstrated a common canaliculus entering the lacrimal sac, and only one demonstrated two separate orifices in the sac (0.08%) [4]. Such a high prevalence of common canaliculus in our study (19/19, 100%) demonstrated again that common canaliculus is the most common type of canaliculus entering a lacrimal sac. The absence of other types of canaliculus opening to a lacrimal sac may be due to our small sample size.

As mentioned above, it is certain that most people have a common canaliculus. The controversy is over whether or not a mucosal fold, or a real valve, exists at the canaliculus entrance into the lacrimal sac. Some studies showed the existence of a valve-like mucosal fold at the junction of the canalicular and lacrimal sac [1, 4, 5]. Aubaret credited Rosenmüller with first describing an irregular mucosal fold located at the superior junction of the canalicular and lacrimal sac in 1797 [1]. Zoumalan et al. observed that 59.7% (74 of 124) had some variation in canalicular/lacrimal sac folds; the remaining 40.3% (50 of 124) had no visible mucosal fold. They found six types of mucosal folds at the canaliculus entrance into the lacrimal sac [4]. Kakizaki et al., who examined the movement of the internal canalicular orifice under endonasal endoscope after a DCR, found the internal canalicular orifice closed during eyelid opening, and the orifice opened during eyelid closing [5]. In clinic, it is impossible to cannulate in the patients with acute dacryocystitis and lacrimal sac mucocele; however, when the lacrimal sac is decompressed after a DCR, cannulation can be performed without difficulty. All of the above phenomena suggested that a valve or mucosal fold may exist at the common canalicular entrance.

However, some researchers demonstrated that no mucosal fold/valve existed at the entrance of the lacrimal canaliculus [1–3, 5, 10]. As mentioned by Kominami et al., there was no histological evidence regarding the real valve in their anatomical and histological study [2]. In their studies, Orhan et al. have not observed the anatomical structure called the Rosenmuller valve [10]. Some studies reported that, during a DCR, the valve structure was not observed at the inner opening of the common canaliculus into the lacrimal sac after careful examination [3, 5]. In 1908, Aubaret reported an experiment by Bert that a filling with colored liquid injected into nasal cavity of cadavers resulted in reflux at the puncta in only 3 of 18 specimens. This functional barrier was attributed to the Hasner valve because the injection of fluid directly into the nasolacrimal canal beyond this region resulted in reflux in all cases. He concluded that once fluid passed the Hasner valve in a retrograde direction, the other valves offered no physiological obstacle to reflux of fluid and were merely inconsistent folds in the mucous membrane [1].

Other studies illustrated that those valve-like functions may be related to the specific configuration of the lacrimal system [3, 13–15]. Tucker et al. used rigid plastic casts of the lacrimal duct system of human cadavers to demonstrate that the common canaliculus has a consistent bend from a posterior direction to an anterior direction and travels anteriorly to enter the sac at an acute angle [3]. They suggested that the anterior angulation of the common canaliculus as it enters the sac may explain the valve-type canalicular obstruction. Enlargement of the sac may result in the narrowing of the acute angle between the common canaliculus and sac. They speculated that the lateral expansion of the sac tends to kink the common canaliculus, thus preventing a reduction in the sac [3, 13]. Kakizaki et al. examined the length and diameter of the intrasac portion of the lacrimal canaliculus in 14 eyelid and orbital specimens from 10 cadavers. They stated that the long length and small diameter of the intrasac lacrimal canaliculus presumably contribute to it acting as an autonomic functional valve at the common internal ostium [14].

Our observation in EE-DCR patients showed that 62.5% of cases had a mucosal fold around the orifice. The mucosal fold can be moved like a valve during the eyelid closing or opening. We have observed that an orifice opened spontaneously with the mucosal fold forward and concurrently with tears or air bubbles flowing into the lacrimal sac when the eyelid closed or Horner's muscles contracted (Figures 1(b) and 1(c)). However, when the eyelid opened or Horner's muscles relaxed, the mucosal fold in the circle area returned back concurrently with the orifice closing (Figure 1(d)). It suggests that the mucosal fold may participate in the tears flowing into the lacrimal sac. The absence of a mucosal fold in 6 out of 16 patients (37.5%) implied that, excepting mucosal fold, other factors, such as Horner's muscles, lacrimal canaliculus, or both of them, may also play a critical role in the functional drainage. The mucosal fold may be involved in the formation of the canalicular negative pressure at the moment of orifice closing or eyelid opening. Similar to Zoumalan's report [4], we also found various types of mucosal folds (Figures 1(a) and 1(c)). We speculated that the types of mucosal folds might be formed by a common canaliculus entering into a lacrimal sac or by the attachment or movement of the muscles around the common canalicular entrance. Although the types of mucosal folds are likely affected by various factors, such as surgery-related mucosal scarring (flattening and stretching in the mucosa) or viewing angle of the nasal endoscope, our results in vivo were similar to Zoumalan's results in cadavers.

The anatomical study showed 47.4% (9/19) of visible mucosal folds in the cadaver samples, which was less than 62.5% of our EE-DCR patients, as well as 59.7% of the report by Zoumalan et al. [4]. Excepting the methods and standards of observation [2, 4, 16], the difference in the prevalence of mucosal folds may be partly attributed to the variance of samples or sample sizes. The anatomical study illustrated that a mucosal fold was inside a circle area, around the orifice (Figures 2(a), 2(c), and 2(e)). This circle area may be consistent with the protruding area in the EE-DCR patient during an eye blink (Figure 1(c)). We also found that the orifice can be opened when we used forceps to relax the muscles around the lacrimal sac and closed when the muscles were stretched (Figures 2(a)–2(f)). The muscles around the lacrimal sac are mainly the branches of the orbicularis oculi muscles or Horner's muscles [17, 18]. Using forceps to imitate the contraction and relaxation of the muscles is like the blink of an eye. The difference in orifice open/close responding to the relaxation/contraction of related muscles, between in vivo and cadavers studies, is likely due to the lacrimal sac of the cadaver losing its normal intrasac tension; thus, the orifice is closed rather than opened when the muscles around the lacrimal sac are stretched. Our in vivo and cadavers studies further proved that there is a mucosal fold at the common canalicular entrance, even if it has not been found in all subjects.

The histological study confirmed that those mucosal folds located at one side of an orifice (Figure 3(a)), with many bundles of circular and longitudinal muscles around this area (Figure 3(b)). Our study is the first description of the mucosal fold existing at the common canalicular entrance histologically. The bundles of muscles may become part of a protruding circle area and may be involved in the movement of a mucosal fold or an orifice. The protruding mucosal fold is likely to be able to cover the orifice when it is moved by related muscles or intrasac pressure (Figure 3(a)). The mucosal fold lined with double-layered columnar epithelial cells implied that it originated from the lacrimal sac and not from the common canaliculus, which may respond to an eye blink more quickly and flexibly.

From the above studies, we supposed that a mucosal fold existing at the junction of a canaliculus entrance into a lacrimal sac may help to maintain the pump function of the lacrimal drainage system under normal circumstances. In particular situations, such as acute dacryocystitis and lacrimal sac mucocele, in addition to an extremely enlarged lacrimal sac, or common canaliculus at an acute angle, alterations of mucosal folds may also be involved in the pathogenesis of those disorders. Thicker or larger mucosal folds due to acute dacryocystitis or lacrimal sac mucocele may interfere with the movement of both mucosal folds and orifices, eventually causing a blockage at the common canalicular entrance. Therefore, mucopurulent discharge inside a lacrimal sac cannot be pressed over to the puncta, or cannulation cannot be performed due to the closed mucosal fold. Perhaps an intraoperative injured mucosal fold, even with a successful EE-DCR, may induce a recurrent epiphora postoperatively due to its inability to close or open. However, those theories need to be further investigated in future clinical studies.

## 4. Conclusions

This study confirmed that common canaliculus is the most common type of canaliculus open to the lacrimal sac. It demonstrated that the mucosal folds existed in a certain ratio, and can be opened/closed when the related muscles are contracted or relaxed. CLS-MFs may help to draw tears flowing into the lacrimal sac under normal circumstances. Alterations of mucosal folds may be involved in the pathogenesis of lacrimal system disorders, such as acute dacryocystitis or lacrimal sac mucocele.
